# Neuromodulatory Effects of Hesperidin in Mitigating Oxidative Stress in Streptozotocin Induced Diabetes

**DOI:** 10.1155/2014/249031

**Published:** 2014-06-19

**Authors:** Mohammad Ashafaq, Laxmi Varshney, Mohammad Haaris Ajmal Khan, Mohd. Salman, Mehar Naseem, Saima Wajid, Suhel Parvez

**Affiliations:** ^1^Department of Medical Elementology and Toxicology, Jamia Hamdard (Hamdard University), New Delhi 110 062, India; ^2^Department of Biotechnology, Jamia Hamdard (Hamdard University), New Delhi 110 062, India

## Abstract

Oxidative stress has been implicated in pathogenesis of streptozotocin- (STZ-) induced diabetes mellitus and its complication in central nervous system (CNS). Recent studies have provided insights on antioxidants and their emergence as potential therapeutic and nutraceutical. The present study examined the hypothesis that hesperidin (HP) ameliorates oxidative stress and may be a limiting factor in the extent of CNS complication following diabetes. To test this hypothesis rats were divided into four groups: control, diabetic, diabetic-HP treated, and vehicle for HP treatment group. Diabetes mellitus was induced by a single injection of STZ (65 mg/kg body weight). Three days after STZ injection, HP was given (50 mg/kg b.wt. orally) once daily for four weeks. The results of the present investigation suggest that the significant elevated levels of oxidative stress markers were observed in STZ-treated animals, whereas significant depletion in the activity of nonenzymatic antioxidants and enzymatic antioxidants was witnessed in diabetic rat brain. Neurotoxicity biomarker activity was also altered significantly. HP treatment significantly attenuated the altered levels of oxidative stress and neurotoxicity biomarkers. Our results demonstrate that HP exhibits potent antioxidant and neuroprotective effects on the brain tissue against the diabetic oxidative damage in STZ-induced rodent model.

## 1. Introduction

Diabetes mellitus (DM) is a chronic disorder of glucose metabolism caused by impaired secretion of insulin from pancreatic *β*-cells, which affects the central and peripheral nervous systems [1]. Globally its incidence is considered to be about 5% of the total population [2]. The chronic hyperglycemia of DM is coupled with eternal damage, dysfunction, and failure of various organs, especially the eyes, kidneys, nerves, heart, and blood vessels [3]. It has been reported that long-term complication of diabetes includes initiation of degenerative processes that cause damage of brain and nerve tissues. Subsequently, it may be associated with cognitive decline and increased risk of dementia [4]. The mature, healthy mammalian brain utilizes glucose only as a source of biological energy as ATP to meet its functional and structural requirements. Glucose consumption is impaired in the brain during diabetes, providing a potential mechanism for increased vulnerability to acute pathological events [5].

There has been a substantial body of evidence suggesting that oxidative stress is a key mechanism in pathogenesis of DM [6]. Oxidative stress results from an imbalance between prooxidant and antioxidant defense systems that are associated with an excessive production of activated oxygen species, including superoxide radical, hydrogen peroxide, and hydroxyl radical or a reduction of endogenous antioxidant defense mechanisms that fail to protect against oxidative damage. The brain is highly vulnerable to the damage caused by free radicals because of its rapid oxidative metabolic activity, high polyunsaturated fatty acids content, relatively low antioxidant capacity, and inadequate neuronal cells repair activity. Therefore brain tissues are predominantly susceptible to oxidative damage induced by diabetes. Cells have enzymatic and nonenzymatic scavenger systems against these free radicals. However, if free radical production and scavenger systems somehow become unbalanced, cells are exposed to oxidative damage resulting in cell injury [7]. In DM, protein glycation, mitochondrial dysfunction, and glucose autoxidation may generate free radicals, which mediate damage to cell structures, including lipids, membranes, proteins, DNA, and disrupt cellular integrity [8].

There is a paucity of literature for understanding deleterious mechanisms involved in diabetes and its complication. Rodent model of Streptozotocin- (STZ-) induced diabetes has been developed to decipher the mechanisms associated with diabetes and to study the potential efficiency of prophylactic/therapeutic strategies for its treatment. STZ is a glucosamine-nitrosourea compound that has been widely used for inducing type-1 diabetes in a variety of animals by affecting degeneration and necrosis of pancreatic *β*-cells [9]. Oxidative damage is implicated in the etiology of diabetes complications; therefore, development of therapeutic agents with antioxidants is preferred.

Hesperidin (HP, 3′,5,7-trihydroxy-4′-methoxy-flavanone-7-rhamnoglucoside), a member of the flavanone group of flavonoids, largely isolated from citrus fruits is known to exhibit antioxidative, anti-inflammatory [10], antihypercholesterolemic [11], and antihyperglycemic [12] activities. The molecular mechanisms of these effects may include scavenging peroxynitrite radicals and inhibition of ROS generation, including hydroxyl radical [13]. However, little information is known about its effect on antidiabetic properties associated with diabetic complications. The above mentioned properties of HP warrants investigating the neuromodulatory potential of HP in a rat model of STZ-induced diabetes and to understand the complex mechanism behind diabetic complications for developing sensible therapeutic strategy.

## 2. Materials and Methods

### 2.1. Materials

Benzylaminehydrochloride (BAHC), bovine serum albumin (BSA), butylated hydroxy toluene (BHT), 1-chloro-2, 4-dinitrobenzene (CDNB), 5,5′-dithiobis (2-nitrobenzoic acid) (DTNB), epinephrine, oxidized glutathione (GSSG), reduced glutathione (GSH), hydrogen peroxide (H_2_O_2_), nicotinamide adenine dinucleotide phosphate (NADPH), o-phosphoric acid (OPA), thiobarbituric acid (TBA), and trichloroacetic acid (TCA) were purchased from Sigma Chemicals Co. (St. Louis, MO, USA). 1-amino-2-naphthol,4-sulfonic acid (ANSA), 2,4-dinitrophenyl hydrazine (DNPH), ethylenediaminetetraacetic acid (EDTA), and sulfosalicylic acid (SSA) were purchased from Merck Limited (Mumbai, India). Guanidine hydrochloride and sodium azide were obtained from Hi-Media Labs (Mumbai, India). STZ and HP were obtained from Sigma Aldrich (St. Louis, USA).

### 2.2. Animal Treatment

Male Wistar rats (250–300 g body weight) were obtained from the animal house of Jamia Hamdard University. Rats were housed in groups of four animals per cage at an ambient temperature of 25 ± 2°C and relative humidity of 45–55% with 12 h light/dark cycles and had free access to standard rodent pellet diet and water* ad libitum*. Experiments were conducted in accordance with the Animal Ethics Committee of the University.

### 2.3. Experimental Design and Induction of STZ Model of Diabetes

To evaluate the protective effect of HP against STZ induced neurotoxicity under* in vivo* conditions, the animals were divided into four groups each having six animals. The first group served as control and saline was given orally, the second was STZ treated (65 mg/kg b.wt. by single* i.p.* injection 3 days before HP treatment to induce diabetes), the third group was STZ treated with HP (50 mg/kg b.wt. orally) for four weeks and the fourth group was pretreated for four weeks with HP alone (50 mg/kg, orally). The doses were selected on the basis of previous study and literature reports [14]. The development of hyperglycemia in rats was confirmed by blood glucose evaluation. The animals that maintained blood glucose significantly higher than control were considered diabetic and were selected for studies. After the 31-day experiment, all rats were sacrificed by cervical dislocation and the brains were excised for further investigation of biochemical estimations.

### 2.4. Homogenate and Postmitochondrial Supernatant (PMS) Preparation

The brain tissue was homogenized in 0.1 phosphate buffer, pH-7.4, to obtain 10% homogenate using a Potter-Elvehjem homogenizer giving 6–8 strokes at medium speed keeping the sample under ice. Homogenate was subjected to differential centrifugation in refrigerated centrifuge at a temperature of 4°C. It was centrifuged at 10,000 rpm for 20 minutes. The resulting pellet is the primary mitochondrial pellet and the supernatant is 10% postmitochondrial supernatant. PMS was used for the estimation of various biochemical analyses.

### 2.5. Biochemical Estimation

#### 2.5.1. Oxidative Stress Markers

*(1) Estimation of Thiobarbituric Reactive Species (TBARS) Content as a Marker of Lipid Peroxidation (LPO).* The assay of LPO was according to the method of Mihara and Uchiyama [15]. The tissue was homogenized in chilled 0.1 M phosphate buffer. 0.250 mL of homogenate was mixed with 0.025 mL of BHT. 1 mL TBA was added to the mixture and after vortexing 3 mL of OPA was added. The resultant mixture was incubated at 90°C for 45 minutes. For blank measurement, no biological sample was taken. The boiling tubes were removed and cooled at room temperature. The absorbance of each supernatant was measured at 535 nm. The rate of LPO was expressed as *μ*moles of TBARS formed/h/g tissue using a molar extinction coefficient of 1.56 × 10^5^ M^−1 ^cm^−1^.

*(2) Estimation of Protein Carbonyl (PC).* Protein carbonyl content was measured according to the method described by Levine et al., [16] as modified from Floor and Wetzel [17]. The PMS (0.5 mL) was reacted with 0.5 mL of 10 mM DNPH in 2 M hydrochloric acid for 1 h at room temperature and precipitated with 0.5 mL of 6% TCA. The protein in the pellet was washed thrice by resuspension in 1.0 mL ethanol/ethyl acetate (1 : 1). Proteins were then solubilized in 0.5 mL of 6 M guanidine hydrochloride and 0.5 mL of 50% formic acid and then centrifuged at 16,000 g for 5 min to remove any trace of insoluble material. The carbonyl content was measured spectrophotometrically at 340 nm. The results were expressed as nmoles of DNPH incorporated/mg protein based on the molar extinction coefficient of 22,000 M^−1 ^cm^−1^.

#### 2.5.2. Nonenzymatic Antioxidants

*(1) Determination of Nonprotein Bound Thiol (NP-SH).* Nonprotein bound thiol (NP-SH) was determined in the tissue sample by using the method of Sedlak and Lindsay [18] as modified by Govil et al., [19]. The reaction mixture consisted of 0.4 mL H_2_O, 0.5 mL PMS (10%), and 0.1 mL of TCA (40%). It was centrifuged at 2,000 rpm for 10–15 min. 0.5 mL of the supernatant was taken with 1 mL Tris buffer (0.4 M, pH 8.9) and 0.025 mL DTNB. The molar extension coefficient of 13,100 M^−1 ^cm^−1^ was used for determination of thiol content. The absorbance was read at 412 nm. 0.9 mL H_2_O and 0.1 mL TCA were taken as blank.

*(2) Determination Reduced Glutathione (GSH*). GSH was determined by the method of Jollow et al. [20]. The reaction is based on the fact that the thiol group of GSH reacts with the–SH reagent (DTNB) to form thionitrobenzoic acid. The PMS was mixed with 4% SSA. It was then incubated at 4°C for a minimum time period of 1 h and then centrifuged at 4°C at 1,200 g for 15 min. The reaction mixture contained 0.1 M phosphate buffer (pH 7.4), 10 mM DTNB, and 0.4 mL mitochondrial sample prepared from brain tissue. The yellow color developed was read immediately at 412 nm on the spectrophotometer. The reduced glutathione concentration was calculated as *μ*moles GSH/mg protein using a molar extinction coefficient of 1.36 × 10^4^ M^−1 ^cm^−1^.

#### 2.5.3. Enzymatic Antioxidant Assays

*(1) Estimation of Glutathione-S-Transferase (GST) Activity.* GST activity was measured by the method of Habig et al. [21] with some modifications. For GST activity measurement, the reaction mixture contained 1.575 mL of 0.1 M sodium phosphate buffer (pH 7.4), 0.2 mL of 10 mM GSH, 0.025 mL of 10 mM CDNB, and 0.2 mL sample in the total volume of 2 mL. The enzyme activity was calculated as nmoles of CDNB conjugate formed/min/mg protein using a molar extinction coefficient of 9.6 × 10^3^ M^−1 ^cm^−1^ at 340 nm.

*(2) Estimation of Glutathione Reductase (GR) Activity.* GR activity was measured by the method of Carlberg and Mannervik [22]. The reaction mixture of 2 mL consists of phosphate buffer (0.1 M, 7.4 pH), 1 mM GSSG, 0.5 mM EDTA, 0.1 mM NADPH, and 10% PMS. The enzyme activity was calculated as NADPH oxidized/min/mg protein using molar extinction coefficient of 6.22 M^−1 ^cm^−1^. GR activity was measured kinetically at 340 nm.

*(3) Estimation of Xanthine Oxidase (XO).* The activity of XO was assayed by the method of Stripe and Della Corte [23]. The reaction mixture consisted of 0.2 mL PNS supernatant, which was incubated for 5 min at 37°C with 0.1 M phosphate buffer (pH 7.4). Then 0.15 mM xanthine was added to the reaction mixture and kept at 37°C for 20 min, which was followed by the addition of 10% PCA and double-distilled water in a total volume of 4 mL. The mixture was then centrifuged at 1500 g for 10 min and the OD was taken at 290 nm. The enzyme activity was calculated as nmoles of uric acid formed/min/mg protein, using a molar extinction coefficient of 12,200 M^−1 ^cm^−1^.

#### 2.5.4. Neurotoxicity Markers

*(1) Estimation of Acetyl Cholinesterase (AChE) Activity.* AChE was estimated by using the method developed by Ellman et al. [24]. The artificial substrate provided, acetylcholine (ATC), is broken down in the presence of AChE to release thiocholine, which reacts with the –SH reagent DTNB to form thionitro benzoic acid. The reaction mixture consists of 2.6 mL phosphate buffer (0.1 M, pH 7.4), 0.4 mL PMS, 0.1 mL DTNB, and 0.02 mL ATC to make a final volume of 3.12 mL. The enzyme activity was calculated as nmoles of ATC hydrolyzed/min/mg protein using a molar extinction coefficient of 1.36 × 10^4^ M^−1 ^cm^−1^.

*(2) Estimation of Na* ^*+*^ */K* ^*+*^ *ATPase Activity.* Na^+^/K^+^ ATPase activity was measured as the release of inorganic phosphate (P_*i*_) by the method of Saleem et al. [25]. The PMS was prepared in 0.2 M Tris-HCl buffer (pH 7.4). The reaction mixture consisted of the following: KCl (0.2 M) 0.2 mL, MgCl_2_ (0.1 M) 0.1 mL, NaCl (1 M) 0.2 mL, tissue 0.1 mL, and Tris-HCl 1.2 mL. The mixture was incubated at room temperature for 5 min and then 0.2 mL of 0.025 M ATP was added to the experimental sample to start the reaction. The mixture was again incubated at 37°C for 15 min. 1 mL of 10% TCA was added to both the reaction mixtures to stop the reaction. Centrifugation was carried out at 3000 rpm for 10 min. The pellet was discarded and the supernatant was taken 0.5 mL of the supernatant, 3.5 mL of distilled water, 0.5 mL ammonium molybdate and 0.5 mL of ANSA were taken to make a final volume of 5 mL. The mixture was incubated at room temperature for 30 min and the OD was taken at 660 nm. The activity was measured as *μ*g P_*i*_ liberated/min/mg protein.

*(3) Estimation of Monoamine Oxidase (MAO) Activity.* MAO was measured by using the method developed by Holt et al., [26], based on oxidation of BAHC to benzaldehyde. The reaction mixture consisted of 0.4 mL of 0.1 M of phosphate buffer (pH 7.4), 1.3 mL distilled water, 0.1 mL of 0.1 M BAHC, and 0.2 mL of PMS supernatant, which was incubated for 30 min at room temperature. Then 1 mL of 10% PCA was added to the reaction mixture and then centrifuged at 1,500 g for 10 min and OD was taken at 280 nm. The enzyme activity was calculated as *μ*moles of BAHC hydrolyzed/min/mg protein using molar extinction coefficient of 7.6925 M^−1 ^cm^−1^.

### 2.6. Protein Determination

Protein content in sample was estimated by the method of Lowry et al. [27] using Bovine Serum Albumin (BSA) as standard. The reaction mixture contains 0.02 mL PNS, 0.98 mL H_2_O, 5 mL ACR (alkaline copper reagent), and 0.5 mL FCR (Folin-Ciocalteu Reagent). After adding all the reagents, samples were placed in the dark for 30 minutes, and after incubation, OD of samples was recorded at 660 nm against a blank of 1 mL H_2_O, 5 mL ACR, and 0.5 mL FCR.

### 2.7. Statistical Analysis

Results were expressed as mean ± standard error (SE). All data were analyzed using analysis of variance (ANOVA) followed by Tukey's test. Values of *P* < 0.05 were considered as significant. All the statistical analyses were performed using graph pad prism 5 software (Graph Pad Software Inc., San Diego, CA, USA).

## 3. Results

### 3.1. Oxidative Stress Markers

#### 3.1.1. Effect of HP Treatment on TBARS Content in the STZ-Induced Diabetic Rat Brain

**Figure 1:**
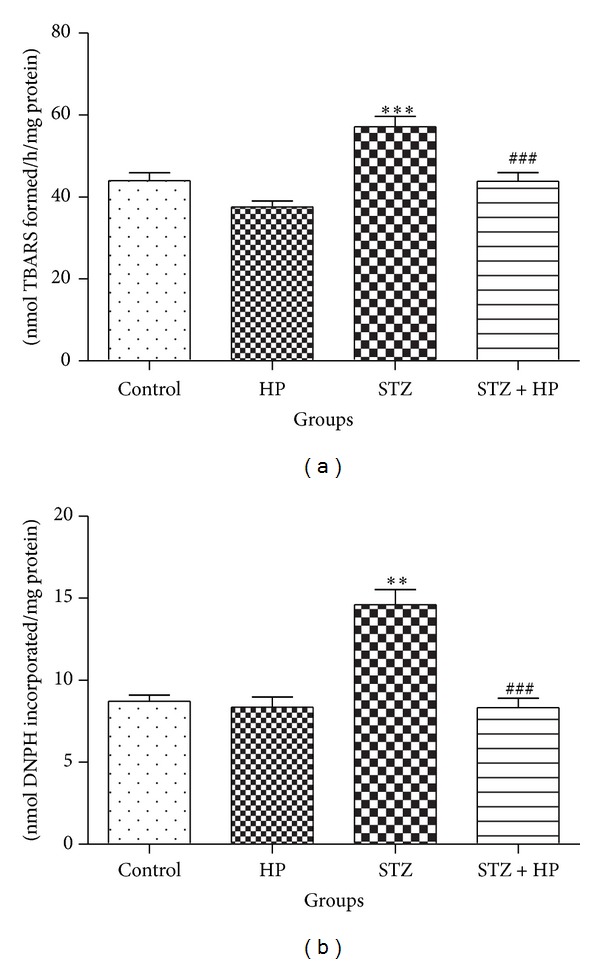
Effect of HP treatment on LPO in terms of (a) TBARS and (b) PC content in the brain homogenate. LPO and PC content were significantly (**P < 0.01 and ***P < 0.001) increased in STZ group rats as compared to control group rats. Hesperidin treatment has decreased the content of LPO and PC significantly in brain (^###^ *P* < 0.001) STZ + HP group as compared to STZ group rats. Each value is represented as mean ± SE (*n* = 6).

The effect of HP on TBARS content was measured to reveal the oxidative damage to lipids in brain of STZ group rats. A significant increase (*P* < 0.001) in TBARS content was observed in the STZ group as compared with control group. HP treatment significantly (*P* < 0.001) decreased TBARS content in the STZ + HP group rats as compared with the STZ group rats. The HP-alone-treated group showed no significant changes in TBARS content as compared with the control group rats (Figure 1(a)).

#### 3.1.2. Effect of HP Administration on PC in the STZ-Induced Diabetic Rat Brain

Protein oxidation was assessed by the determination of PC content. STZ induced a significant (*P* < 0.01) increase in PC content in STZ group rats. HP treatment significantly (*P* < 0.001) reduced the PC content in the STZ + HP group rats as compared with the STZ group rats. There was no difference in PC content in rats treated with HP alone when compared with control group (Figure 1(b)).

### 3.2. Nonenzymatic Antioxidant Status

#### 3.2.1. HP Treatment Protected the NP-SH Level in the STZ-Induced Diabetic Rat Brain

**Figure 2:**
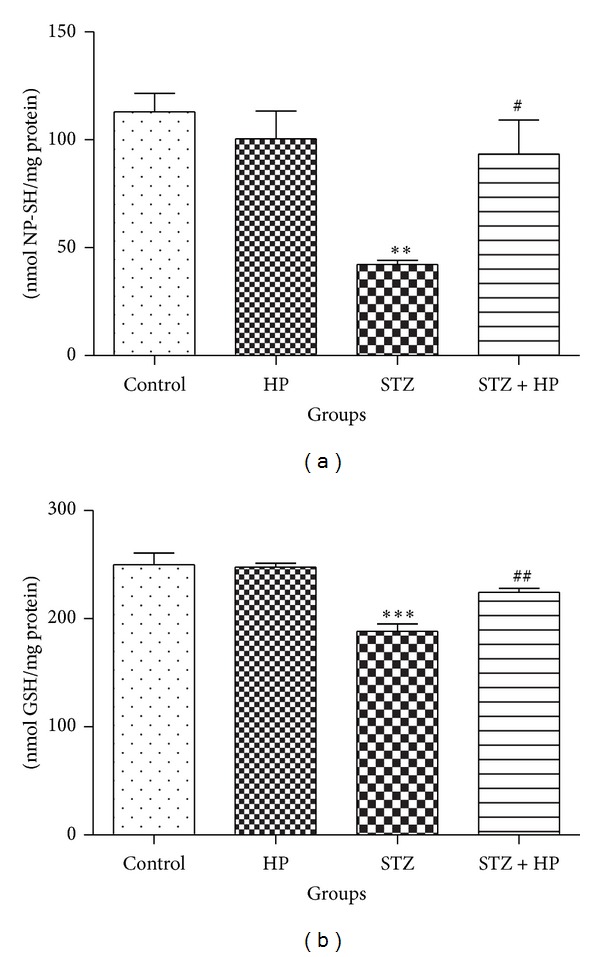
Effect of HP treatment on (a) NP-SH and (b) GSH level in rat brain. Each value is represented as mean ± SE (*n* = 6). NP-SH and GSH was significantly decreased in (**P < 0.01 and ***P < 0.001) STZ group rats as compared to control group. HP treatment has significantly (^#^ *P* < 0.05 and ^##^ *P* < 0.01) increased the level of NP-SH and GSH in STZ + HP group as compared to STZ group rats. Each value is represented as mean ± SE (*n* = 6).

A significant decrease (*P* < 0.01) in the level of NP-SH was observed in STZ group rats when compared with control group. HP treated STZ group rats have shown a significant (*P* < 0.05) increase in NP-SH level when compared with STZ alone group. HP alone group showed no significant changes in NP-SH level as compared to control (Figure 2(a)).

#### 3.2.2. HP Treatment Restored GSH Level in the STZ-Induced Diabetic Rat Brain

Protective effect of HP on GSH was observed in the rat brain. The level of GSH was depleted significantly (*P* < 0.001) in the STZ group rats as compared to control group. Hesperidin treatment has protected its level significantly (*P* < 0.01) in STZ + HP group as compared to STZ group. HP alone treated group exhibited no significant change in GSH level as compared to control group (Figure 2(b)).

### 3.3. Enzymatic Antioxidant Activities

#### 3.3.1. HP Supplementation Attenuated the Activities of Antioxidant Enzymes

**Figure 3:**
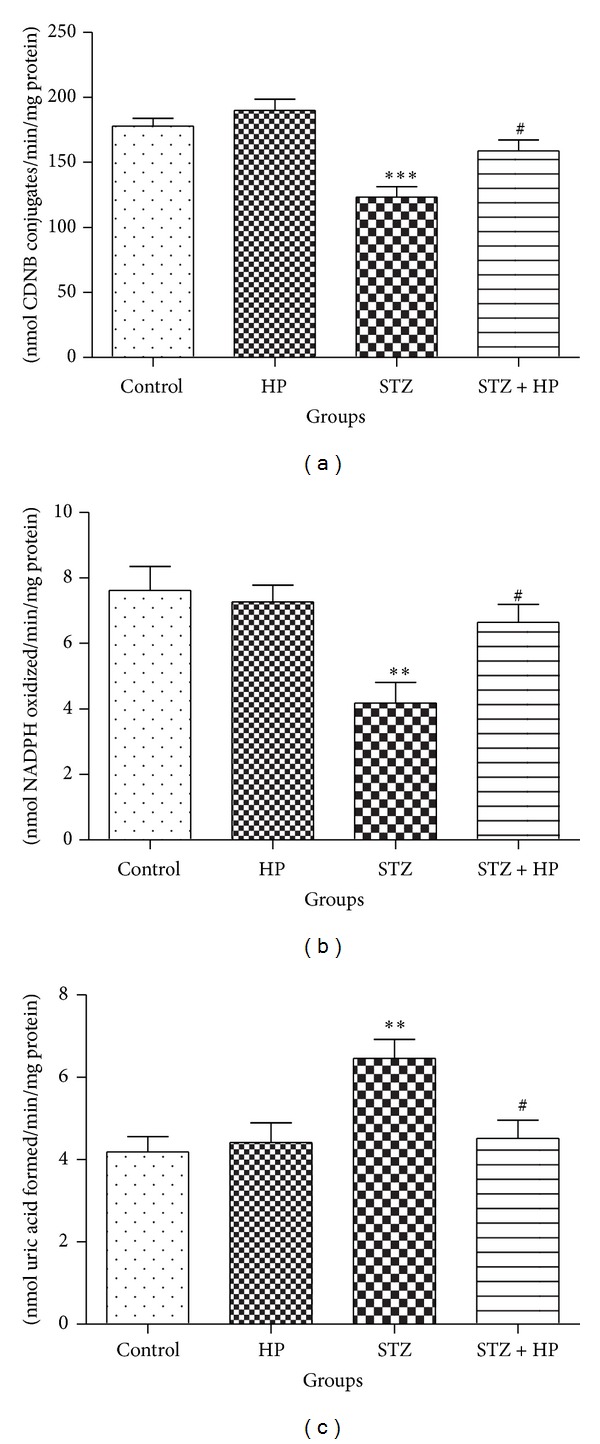
STZ induced significant (**P < 0.01 and ***P < 0.001) alterations in the activities of antioxidant enzymes, (a) GST, (b) GR, and (c) XO, in the STZ group rats when compared with the control group rats. HP treatment protected the activity of (a) GST and (b) GR in rat brain. Administration of HP has significantly attenuated the activity of GST and GR (^#^ *P* < 0.05) in the STZ + HP group rats as compared with the STZ group rats. The activity of XO was significantly (**P < 0.01) increased in STZ group as compared to control group rats. Administration of HP has significantly (^#^ *P* < 0.05) lowered the activity of XO in the STZ + HP group rats as compared with the STZ group rats. Each value is represented as mean ± SE (*n* = 6).

The activities of antioxidant enzymes (GST, GR, and XO) were decreased significantly (*P* < 0.01–0.001) in the brain of STZ group rats when compared with the control group. The activities of these enzymes were restored significantly (*P* < 0.05) in the HP supplemented STZ treated group rats (STZ + HP) when compared with STZ group. No significant change was observed in the HP-treated control group rats as compared with the control group (Figures 3(a), 3(b), and 3(c)).

### 3.4. Neurotoxicity Biomarkers

#### 3.4.1. Effect of HP on AChE, Na^+^-K^+^ ATPase, and MAO Activity on STZ-Treated Rats

**Figure 4:**
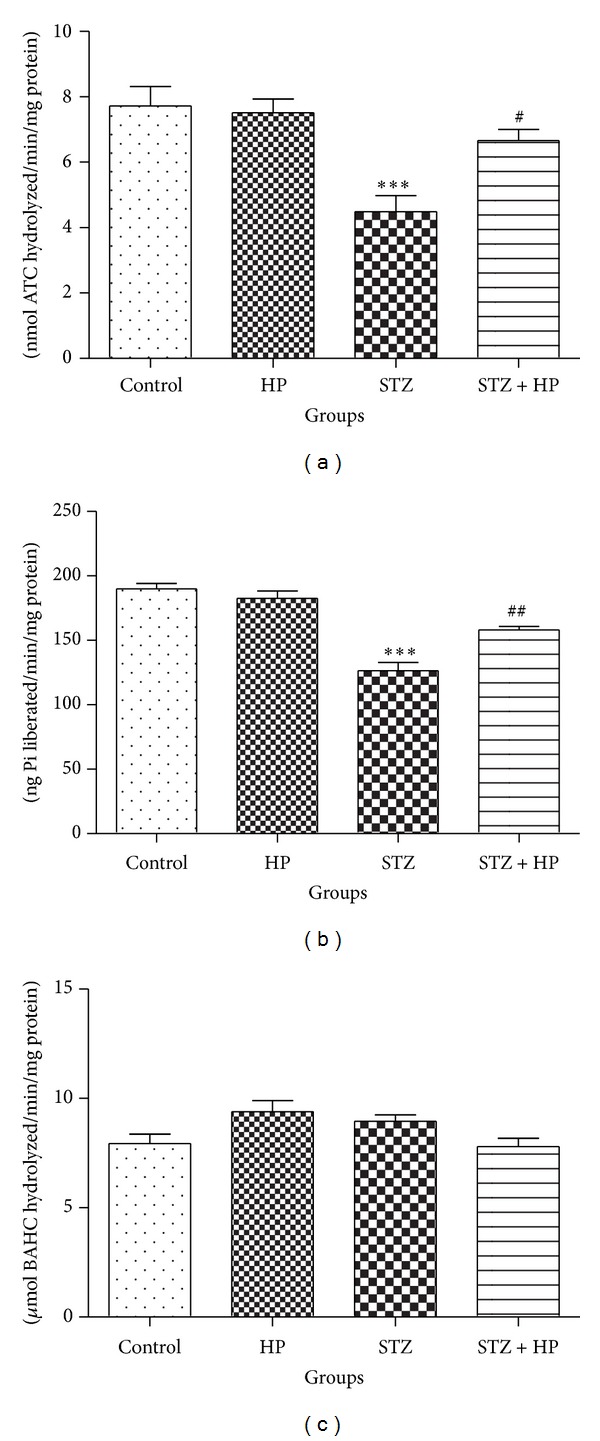
A significant (***P < 0.001) alteration was observed in the activity of neurotoxicity marker enzymes (a) AChE and (b) Na^+^/K^+^-ATPase in the STZ group rats as compared with the control group. Administration of HP has significantly (^#^ *P* < 0.05 and ^##^ *P* < 0.01) attenuated the activity of these enzymes in the STZ + HP group rats as compared with STZ group rats. No significant changes in the activity of (c) MAO in STZ as well as STZ + HP group rats were observed in the rat brain. Each value is represented as mean ± SE (*n* = 6).

The activity of AChE was decreased significantly (*P* < 0.001) in STZ group as compared to control group and treatment with HP has significantly (*P* < 0.05) increased the activity of AChE in the HP + STZ group rats. No significant change was observed between the hesperidin treated control groups and control group (Figure 4(a)). The activity of Na^+^-K^+^ ATPase was significantly decreased (*P* < 0.001) in STZ group as compared to control group rats. HP supplementation significantly attenuated (*P* < 0.01) the activity of Na^+^-K^+^ ATPase in STZ + HP group as compared to STZ group rats. There was no significant alteration in the activity of Na^+^-K^+^ ATPase in HP alone treated group when compared to control group rats (Figure 4(b)). There was no significant change in the activity of MAO in control, HP treated, and STZ treated rats when compared between the groups (Figure 4(c)).

## 4. Discussion

Experimental animal models of diabetes exhibit abrupt generation of free radical and oxidative stress due to persistent and chronic hyperglycemia. The current study examined a single dose of HP in attenuating oxidative stress and associated biochemical alterations in STZ-induced diabetic rat brain. We have observed that the treatment with HP significantly restores endogenous enzymatic and nonenzymatic antioxidants, suppresses the oxidative stress indices, and reinforces the neurotoxicity biomarkers ameliorating oxidative stress.

Evidence has accumulated showing the massive generation of reactive free radicals in diabetes and the resulting increase in LPO and protein oxidation which leads to cellular disintegrity of neuron [28, 29]. Lipid peroxides and protein oxidation are the secondary products of oxidative stress causing secondary injury by further generating relatively more stable and diffusible cytotoxic agents such as malondialdehyde and 4-hydroxy-trans-2-nonenal, respectively, and magnifying oxidative stress [30]. In the present study, the level of LPO and protein oxidation was significantly increased in the diabetic rats, which was significantly decreased by the HP treatment. These data are consistent with the idea that hyperglycemia induces oxidative stress in animal models, as shown earlier in [31].

Biochemical analyses of brain homogenate show significant reduction of GSH and NP-SH which are natural reservoirs of the reductive capacity of the cell [32]. Increasing evidence shows that hyperglycemia due to DM leads to oxidative stress in the central nervous system. The potential harmful effects of ROS are controlled by antioxidant defense mechanisms such as GSH. GSH is a well-known nonenzymatic endogenous antioxidant against ROS in the cellular defense system. The low levels of GSH may be directly related to increased ROS, lipid peroxides, and highly reactive hydroxyl radicals [33]. The detoxification pathway of ROS involves oxidation of GSH to glutathione disulfide (GSSG), resulting in a decrease of GSH level. Reduction of tissue GSH and NP-SH content enhances the cellular damage caused by oxidative stress. A possible reason for the elevated LPO in STZ induced diabetic group may be the reduction of GSH and NP-SH levels. We have observed depleted GSH and NP-SH levels in diabetic rat brain and pretreatment with HP restored the depleted levels of GSH and NP-SH which is in corroboration with the previous observations where antioxidants were used as a remedy in experimental STZ rat models [10, 32, 34].

Furthermore, the activities of the key endogenous antioxidant enzymes are decreased during the oxidative stress induced by STZ [35, 36]. The concentrations of the ROS are modulated by antioxidant enzymes such as GST and GR [37]. As all antioxidant defenses are interconnected, disruption of one would disrupt the whole microenvironment. GST catalyzes the detoxification of oxidized metabolites of catecholamine (o-quinone) and may serve as an antioxidant system preventing degenerative cellular process [38], whereas GR is found in many tissues, and it enables the cell to sustain adequate levels of cellular GSH. The overproduction of free radicals in STZ diabetic rat brain which, in turn, causes oxidative damages to membrane's lipid and protein, and ultimately leads to a decrease in the content of GSH and activity of its dependent enzyme (GR and GST) [39]. However, our finding suggests that activity of antioxidant enzymes (GR and GST) significantly depleted in the brain of diabetic group rats which was significantly counteracted by the alteration in the markers of oxidative damage after treatment with HP. Previous studies have indicated that HP possesses scavenging and neuroprotective properties [40–42].

Superoxide radical generation and increased XO activity have been implicated in brain tissues of STZ-induced experimental diabetes in rat, which may be responsible in the pathogenesis and complications of DM [43]. XO which could be formed from xanthine dehydrogenase in pathological conditions is able to catalyze the reduction of oxygen leading to the formation of superoxide where the reaction is considered to play a vital role in the oxidative damages [44]. In contrast, the present findings indicate that XO were increased significantly in the brain of diabetic rat. HP treatment significantly decreases the XO level in HP treated diabetic rat. This was further supported by previous studies, where an increased level of XO activity in diabetic rat brain was observed [45].

A significant alteration in the activity of rat brain enzymes, such as AChE, Na^+^/K^+^-ATPase, and MAO can also provide for measurement of the brain oxidative stress levels induced by STZ [46, 47]. Brain tissue shows a considerable difference in AChE activity in diabetic rat. AChE is a cholinergic enzyme, which promotes the hydrolysis of the neurotransmitter acetylcholine, has a pivotal role in regulating many vital functions, and responds to various insults including oxidative stress, an important event that has been related to the pathogenesis and progression of diabetes [48, 49]. In this study we have observed significant depletion in AChE activity in rat brain tissue and this effect can be reversed by the supplementation of HP. The inhibition of AChE activity by STZ induced oxidative stress in brain tissue results in the accumulation of ACh at cholinergic synapses which leads to the overstimulation of muscarinic and nicotinic receptors, decreases the cellular metabolism, and also induces deformities of cell membrane and disturbs metabolic and nervous activities [50].

Additionally, there was a significant decrease in brain Na^+^/K^+^-ATPase activity in diabetic rats. Oxidative stress triggers membrane dysfunction, increasing membrane permeability to ions by inactivating membrane bound enzymes like Na^+^/K^+^ ATPase, which is very susceptible to free radical attack [51]. Na^+^/K^+^ ATPase plays an important role in maintaining sodium and potassium gradients and the neuronal electrical functions implicated in neuronal metabolic energy production as well as in the uptake and release of catecholamines [46, 52]. Our results strongly suggest that HP may find a role in reducing the complications of neurotoxicity in DM. Future studies should examine the fact that HP therapy targeted to brain toxicity may constitute an interesting strategy to mitigate neurotoxicity at molecular level.
